# Editorial: Statistical approaches, applications, and software for longitudinal microbiome data analysis and microbiome multi-omics data integration

**DOI:** 10.3389/fgene.2025.1624791

**Published:** 2025-05-29

**Authors:** Huilin Li, Jun Chen, Mogens Fenger, Yuan Jiang

**Affiliations:** ^1^ Division of Biostatistics, Department of Population Health, New York University Grossman School of Medicine, New York University, New York, NY, United States; ^2^ Division of Computational Biology, Department of Quantitative Health Sciences, Mayo Clinic, Rochester, MN, United States; ^3^ Department of Clinical Biochemistry, Copenhagen University Hospital Hvidovre, Copenhagen, Denmark; ^4^ Department of Statistics, Oregon State University, Corvallis, OR, United States

**Keywords:** artificial intelligence, longitudinal data analysis, microbiome, multi-omics integration, normalization, sparsity, temporal dynamics, visualization

The microbes inhabiting the human body do not exist in isolation but form an integrated and dynamic microbial community known as the microbiome. This community interacts with nearly every major systemic component of the host and plays a pivotal role in regulating health and disease (Daniel et al., 2021; Ruff et al., 2020). Its modifiable nature makes it an attractive and promising therapeutic target. The emergence of longitudinal microbiome studies has significantly advanced our understanding of microbial stability, resilience, and temporal dynamics. At the same time, innovations in multi-omics technologies—such as transcriptomics, epigenomics, metabolomics, and proteomics—now enable systematic, multilayered profiling of host-microbiome interactions (Wang et al., 2019).

Despite these advancements, analyzing complex, high-dimensional datasets remains a major challenge. Microbiome data, in particular, present unique difficulties due to their compositionality, sparsity, and underlying evolutionary relationships. This Research Topic is curated to address these pressing challenges by highlighting robust statistical methods capable of capturing intricate temporal patterns in longitudinal microbiome data, as well as innovative approaches for uncovering complex biological interrelationships through microbiome multi-omics integration.

Longitudinal microbiome studies offer powerful insights into the dynamics of microbial systems over time. For instance, Palmer et al. introduce a novel analytic framework that identifies time-lagged associations between longitudinal microbial abundance profiles and a final health outcome or disease status. Their method uses group penalization to detect both the direction and duration of these associations, offering a powerful new lens through which to study host-microbiome interactions.

Addressing common pitfalls in microbiome analysis, Shi et al. propose a practical framework that accounts for the skewness and heteroscedasticity of microbiome abundance data. Their approach integrates Poisson (log-linear) regression with robust standard error estimation using the bootstrap method and sandwich estimators, substantially enhancing the statistical inference in differential abundance analysis. Notably, while normalization is a critical preprocessing step, there has been a lack of practical methods specifically designed for longitudinal microbiome data. To fill this gap, Luo et al. present *TimeNorm*, a novel normalization method tailored for time-course microbiome data that accounts for both compositionality and temporal dependency. Through simulation studies and real-world application, they demonstrate that *TimeNorm* significantly improves the power of downstream differential abundance analyses.

Given the sparsity and heterogeneity of microbial species’ temporal patterns, Li et al. introduce *ZINQ-L*, a zero-inflated quantile-based framework for longitudinal microbiome differential abundance testing. This flexible, distribution-free method is well-suited for identifying heterogeneous associations in complex datasets and demonstrates improved testing power, providing a robust and powerful option to the existing methods in the field.

Visualization remains an indispensable component of microbiome data analysis. Litter et al. propose an enhanced framework for visualizing repeated-measures microbiome data using Principal Coordinate Analysis adjusted for covariates via linear mixed models. This approach supports nuanced data exploration and clearer biological interpretation in the presence of confounding factors and complex experimental designs including longitudinal and clustered designs.

Advancements in artificial intelligence, especially deep learning and large language models, are beginning to shape microbiome research. Yan et al. provide a timely review of the applications of AI-driven models in microbiome and metagenomics analysis. Their insights highlight emerging opportunities and set the stage for future AI-empowered discoveries in the field.

This Research Topic also explores multi-omics integration. Chen et al. combine 16S microbiome data with targeted metabolomics to uncover biomarkers relevant to aging-related diseases. Their findings reveal disrupted intestinal barrier function and elevated secondary bile acid metabolites in aging populations, correlated with shifts in specific bacterial taxa. Complementing this work, Deng et al. propose a structure-adaptive canonical correlation framework that integrates microbiome data with other high-dimensional omics layers while respecting compositional constraints and incorporating prior biological structure through adaptive penalization. This method paves the way for deeper insights into microbiome-host interactions across molecular domains such as the genome, transcriptome, epigenome, metabolome, and proteome.

Collectively, the contributions in this Research Topic mark a significant step forward in the development of cutting-edge analytical methodologies tailored for the complexities of longitudinal and multi-omics microbiome research. By addressing key challenges in normalization, sparsity, temporal dynamics, and data integration, these works provide powerful tools and frameworks to uncover deeper insights into the microbiome’s dynamic role in health and disease. We hope this Research Topic will inspire continued methodological innovation and interdisciplinary collaboration, ultimately advancing the field toward more personalized and microbiome-informed healthcare.
